# TRIM52 knockdown inhibits proliferation, inflammatory responses and oxidative stress in IL‐1β‐induced synovial fibroblasts to alleviate temporomandibular joint osteoarthritis

**DOI:** 10.1111/jcmm.18244

**Published:** 2024-03-23

**Authors:** Tie Ma, Chuan‐bin Wu, Qing‐xia Shen, Qiang Wang, Qing Zhou

**Affiliations:** ^1^ School and Hospital of Stomatology China Medical University Shenyang Liaoning China; ^2^ Department of Oral and Maxillofacial Surgery Liaoning Provincial Key Laboratory of Oral Disease Shenyang Liaoning China; ^3^ Department of Stomatology Shengjing Hospital of China Medical University Shenyang Liaoning China

**Keywords:** inflammatory responses, oxidative stress, TLR4/NF‐κB, TMJOA, TRIM52

## Abstract

To explore the mechanism of tripartite motif 52 (TRIM52) in the progression of temporomandibular joint osteoarthritis (TMJOA). Gene and protein expression were tested by quantitative real‐time polymerase chain reaction and western blot, respectively. The levels of pro‐inflammatory cytokines and oxidative stress factors were evaluated using enzyme‐linked immunosorbent assay and biochemical kit, respectively. Cell counting kit‐8 and 5‐ethynyl‐2′‐deoxyuridine assays were carried out to assess cell proliferation. Immunofluorescence was used to detect the expression of CD68 and Vimentin in primary synovial fibroblasts (SFs). Haematoxylin and eosin staining and Safranin O/Fast green were used to evaluate the pathological damage of synovial and cartilage tissue in rats. TRIM52 was upregulated in the synovial tissue and SFs in patients with TMJOA. Interleukin (IL)‐1β treatment upregulated TRIM52 expression in TMJOA SFs and normal SF (NSF), promoting cell proliferation, inflammatory response and oxidative stress in NSF, SFs. Silence of TRIM52 relieved the cell proliferation, inflammatory response and oxidative stress induced by IL‐1β in SFs, while overexpression of TRIM52 enhanced IL‐1β induction. Meanwhile, IL‐1β induction activated toll‐like receptor 4 (TLR4)/nuclear factor (NF)‐κB pathway, which was augmented by upregulation of TRIM52 in NSF, and was attenuated by TRIM52 knockdown in SFs. Besides, pyrrolidinedithiocarbamic acid ameliorated IL‐1β‐induced proliferation and inflammatory response by inhibiting TLR4/NF‐κB signalling. Meanwhile, TRIM52 knockdown inhibited cell proliferation, oxidative stress and inflammatory response in IL‐1β‐induced SFs through downregulation of TLR4. TRIM52 promoted cell proliferation, inflammatory response, and oxidative stress in IL‐1β‐induced SFs. The above functions were mediated by the activation of TLR4/NF‐ κB signal pathway.

## 1 INTRODUCTION

Temporomandibular joint osteoarthritis (TMJOA), also known as temporomandibular joint disorder syndrome, is one of the most common oral‐maxillofacial diseases.^1^ TMJOA is a group of syndromes caused by various reasons, mainly characterized by joint pain, joint clicking, cheek swelling and jaw movement abnormalities.^2^ In the early stage of TMJOA, the synovium manifested a proliferation process accompanied by a large amount of inflammatory cell infiltration. In the late stage, the synovium showed excessive fibrosis, resulting in the aggravation of TMJOA.^3^ TMJOA severely restricted eating and speech, thereby influencing the quality of life. Though efforts have been made to elucidate the pathogenesis of TMJOA, its underlying molecular mechanism is still obscure.

Synovial fibroblasts (SFs), the main cell type of synovial cells, are mainly distributed in the synovial sublining and lining layer.^4^ SFs normally provide the articular cavity and surrounding cartilage with nourishment and lubrication, and maintain the integrity of cartilage.^5^ In TMJOA patients, SFs, the direct effector cells of inflammatory response and matrix remodelling, are activated and secrete matrix proteases and chemokines. Such substances can induce inflammation and immune response, leading to the destruction of cartilage and bone tissue.^6^ Thus, SFs play an important role in the progression of TMJOA. By considering this, SFs are usually used to mimic the pathogenesis of synovium.

Tripartite motif proteins have E3 ubiquitin ligase activity, and are involved in the various cell processes, such as intracellular signal transduction, cell differentiation, apoptosis, inflammation, and antiviral host defences.^7^, ^8^, ^9^ Tripartite motif 52 (TRIM52), located on chromosome 5q35.3, is a member of the tripartite motif family and serves multiple roles in human diseases. For instance, TRIM52 induces the upregulation of nuclear factor (NF)‐κB to promote the growth of human benign prostatic hyperplasia cells by regulating the ubiquitination of tumour necrosis factor receptor‐associated factor.^10^ In addition, TRIM52 has been confirmed as an anti‐viral factor, which can hamper the infection and replication of Japanese encephalitis virus by inducing the proteasome‐dependent degradation of viral nonstructural protein 2A.^11^ TRIM52 is considered a momentous regulator of inflammation. In an in vitro model of periodontitis, downregulation of TRIM52 mitigates lipopolysaccharide‐induced inhibition of proliferation, apoptosis promotion and inflammatory response in human periodontal ligament cells through the toll‐like receptor 4 (TLR4)/NF‐κB signalling.^12^ However, whether TRIM52 participates in the pathogenesis of TMJOA has not yet been investigated.

In this study, we aimed to explore the role and mechanism of TRIM52 in the progression of TMJOA. Our findings indicated that the knockdown of TRIM52 inhibited interleukin (IL)‐1β‐induced proliferation, inflammatory responses and oxidative stress in SFs from patients with TMJOA. Besides, animal experiments also confirmed that knockdown of TRIM52 inhibited synovial hyperplasia and inflammatory infiltration. These findings suggested that TRIM52 was considered a potential therapeutic target for the treatment of TMJOA.

## 2 MATERIALS AND METHODS

### 2.1 Ethics statement

Our study has obtained approval from the ethics committee of the Hospital of Stomatology, China Medical University (2022PS028K), and all the patients were informed and voluntarily signed the informed consent prior to surgery.

### 2.2 Clinical samples

The TMJOA synovium specimens were obtained from the patients with TMJOA, who underwent open temporomandibular joint (TMJ) surgery at the Hospital of Stomatology, China Medical University. All patients without systemic disease ranged in age from 19 to 50, including four females and one male. The normal noninflamed synovium specimens were acquired from three patients with pathologically confirmed condylar hyperplasia undergoing a high condylotomy. The patients with condylar hyperplasia included one male and two females between the ages of 22 and 31.

### 2.3 Primary synovial fibroblasts isolation and culture

The synovium samples were washed three times in phosphate‐buffered saline (PBS; Solarbio, Beijing, China) with the addition of 100 unit/ml penicillin and 100 mg/mL streptomycin. Subsequently, the samples were diced into 1 mm^3^ species and then placed in a 25 mL flask with Dulbecco's Modified Eagle's Medium (DMEM) containing 15% fetal bovine serum (Solarbio) at 5 mm intervals. The flask was maintained in an incubator at 37°C, 5% CO_2_, and the medium was changed every 3 days until the cells reached confluence. The cells were collected and digested with 0.25% trypsin (Solarbio). A 1:2 dilution of the cells was utilized for further incubation, and the third‐generation and fourth‐generation cells were used in the experiment.

We isolated SFs from three normal synovial tissues and five TMJOA synovial tissues. Such tissues were designated as normal synovial fibroblasts (NSF)‐a, NSF‐b, NSF‐c, TMJOA SF‐d, TMJOA SF‐e, TMJOA SF‐f, TMJOA SF‐g and TMJOA SF‐h, respectively. The mixture of TMJOA SF‐d and SF‐e was cultured and named as SF2. Similarly, SF3 was co‐cultured with TMJOA SF‐f, TMJOA SF‐g and TMJOA SF‐h, while NSF was designated by co‐culture of NSF‐a, NSF‐b and NSF‐c. SF2, SF3 and NSF were selected for the following experiments.

### 2.4 Immunofluorescence

SFs were fixed with 4% paraformaldehyde for 10 min, followed by permeabilization with 0.1% Triton™ X‐100 for 10 min. Then the cells were blocked with 1% bovine serum albumin (BSA) for 1 h. Following this, the cells were labelled with a primary antibody against vimentin and CD68 (Abcam, Cambridge, UK), followed by incubation with a fluorescein‐conjugated secondary antibody. The cell nuclear was stained with 4′,6‐diamidino‐2‐phenylindole (DAPI) before the images were captured using a fluorescence microscope.

### 2.5 Cell transfection

The full sequence of TRIM52 or TLR4 was amplified and cloned into the pcDNA3.1 vector to generate pcDNA‐TRIM52 and pcDNA‐TLR4 plasmid. Control siRNA (si‐ctrl or si‐control) and TRIM52 siRNA (si‐TRIM52) were purchased from GeneChem (Shanghai, China) and the sequences were shown in Table S1. NSF was transfected with pcDNA‐TRIM52 or pcDNA‐ctrl (pcDNA3.1 vector control), while SF2 and SF3 were transfected with si‐ctrl or si‐TRIM52 at concentrations of 1 × 10^8^ transducing units (TU)/ml. SFs were transfected with si‐TRIM52 and pcDNA (pcDNA3.1 vector) or pcDNA‐TLR4. Cell transfection was undertaken using Lipofectamine 2000 (Invitrogen, Carlsbad, CA, USA) in accordance with the manufacturer's instructions.

Transfected or non‐transfected SFs were incubated in a medium containing 10 ng/mL IL‐1β or different concentrations of IL‐1β (0, 0.1, 1 and 10 ng/mL). All SFs were collected after 24 h for further testing.

### 2.6 Quantitative real‐time polymerase chain reaction

RNA isolation was conducted using TRIzol reagent (Invitrogen, Carlsbad, CA, USA). Quantitative real‐time polymerase chain reaction (qRT‐PCR) assay was performed to determine the expression of TRIM52 using PrimeScript™ One Step RT‐PCR kit (RR055B, TAKARA, Tokyo, Japan), with β‐actin as an internal control. According to the 2 ^−ΔΔCt^ method, the relative expression of TRIM52 was calculated.

### 2.7 Western blot

After treatment, the cells were collected and lysed with radioimmunoprecipitation assay buffer (P0013B, Beyotime, Shanghai, China). After centrifugation, the supernatants were harvested and tested for protein concentration using a bicinchoninic acid method. 30 μg of total protein was separated using 10% sodium dodecyl sulfate–polyacrylamide gel electrophoresis and then the protein was transferred onto a polyvinylidene fluoride membrane. After blocking with 5% skim milk, the membrane was examined with the primary antibodies (Abcam, Cambridge, MA, USA) against TRIM52, TLR4, p‐IkappaB kinase (IKK), IKK, p65 and β‐actin, followed by immunoblotting with horseradish peroxidase‐conjugated secondary antibodies (Abcam). The blots were developed using an enhanced chemiluminescence kit (Beyotime, Shanghai, China) and quantified with Image J software (National Institutes of Health, Bethesda, MD, USA).

### 2.8 Cell count kit‐8 assay

After treatment, the cells were seeded in 96‐well plates and serum‐starved for 8 h. Following this, the cells were stimulated with IL‐1β (0, 0.1, 1, 10 ng/mL) for 24 h. Afterward, the cells were incubated with cell count kit‐8 (CCK‐8) solution (Solarbio) and the absorbance of each well was measured at a wavelength of 450 nm using a microplate reader.

### 2.9 5‐Ethynyl‐2′‐deoxyuridine assay

Cell proliferation was measured using the Click‐iT EdU Alexa Fluor 594 Imaging Kit (C10339, Invitrogen, Carlsbad, CA, USA). After stimulation, NSF, SF2 and SF3 cells were reacted with 5‐ethynyl‐2′‐deoxyuridine labeling solution (final concentration of 10 μM) for 3 h at 37°C, followed by fixation with 3.7% formaldehyde for 15 min. After incubation with 0.5% Triton X‐100 for 20 min at room temperature, the cells were rinsed with PBS solution and then incubated for 30 min using a Click‐iT reaction cocktail. Subsequently, the cells were stained with Hoechst 33342 from Invitrogen, and the images were acquired employing fluorescence microscopy.

### 2.10 Enzyme‐linked immunosorbent assay

After treatment, the cell culture medium and rat blood were collected and centrifugated at 500 g, 4°C for 5 min to obtain the cell supernatant and serum. The cell supernatant and serum were used to test the levels of interleukin‐6 (IL‐6), tumour necrosis factor‐α (TNF‐α), monocyte chemoattractant protein‐1 (MCP‐1) and vascular endothelial growth factor (VEGF) using human IL‐6 enzyme‐linked immunosorbent (ELISA) kit (ab178013, Abcam, Cambridge, UK), human TNF‐α ELISA kit (ab181421, Abcam), human CCL2/MCP‐1 ELISA kit (ab179886, Abcam) and human VEGF ELISA kit (ab100662, Abcam), following the manual of the manufacturer.

### 2.11 Biochemical detection

The SFs were lysed with western and immunoprecipitation cell lysates and the protein concentrations were determined using a bicinchoninic acid protein concentration assay kit. Malondialdehyde (MDA) content in SFs was then determined according to the instructions of the MDA detection kit (BC0025, Solarbio, Beijing, China). Reactive oxygen species (ROS) levels of SFs were measured according to the ROS test kit (CA1410, Solarbio) instructions.

### 2.12 Construction of TMJOA rat model

Twenty‐five 4‐week‐old female Sprague–Dawley rats, weighing 160–200 g, were purchased from the Latin American and Caribbean Society of Medical Oncology (Shanghai, China). A model of TMJOA was established by unilateral anterior occlusion induction as described previously.^13^ Twenty rats were induced by unilateral anterior occlusion induction and the remaining five rats were sham‐induced as controls. Briefly, a low‐speed handsaw was adopted to cut a No.20 teat cannula into an 8 mm‐length tube. Then one side of the tube was bent at 135° to a length of 3.5 mm. After 1% pentobarbital anaesthesia, this crown was adherent to the left lower incisor of the rats to induce the unilateral anterior occlusion change. The metal crown was checked at least once a day to ensure that it was fixed in place. One week after the initial treatment, the 20 rats induced by the unilateral anterior occlusion were randomly divided into four groups (TMJOA, TMJOA + si‐control, TMJOA + si‐TRIM52 and TMJOA + si‐TRIM52 + pcDNA‐TLR4) with five rats in each group. The si‐ctrl, si‐TRIM52 and pcDNA‐TLR4 were dissolved separately in PBS, and then they (1 × 10^9^ TU) were slowly injected into the left TMJ cavity with 25 μL per rat twice a week for 4 weeks.^14^ The rats in the sham group and TMJOA group were injected with PBS once a week, and the rats were sacrificed after 4 weeks. Besides, the left TMJ and periarticular tissue were collected for haematoxylin and eosin staining and safranin O/fast green (S&F) staining. The inflammatory infiltrate score, a thickening score of the synovial membrane and Mankin score were scored as described previously by two blinded and experienced pathologists.^15^, ^16^ Inflammatory infiltrates were classified into a 0–3 scale, where: 0 = no infiltrate; 1 = discrete infiltration; 2 = moderate synovial infiltrate and 3 = intense synovial infiltration. For thickening of the synovial membrane, a scale of 0–3 was used, where: 0 = no thickening; 1 = discrete thickening; 2 = moderate synovial thickening and 3 = intense thickening of the synovial membrane.

### 2.13 Statistical analysis

Our results were expressed as mean ± standard deviation or standard error of the mean, and were statistically analysed with Student's *t*‐test (two groups) or one‐way analysis of variance (more than two groups) by GraphPad Prism 9 (GraphPad Software, San Diego, CA, USA). *p* < 0.05 was considered statistical significance.

## 3 RESULTS

### 3.1 TRIM52 was upregulated in TMJOA

**FIGURE 1:**
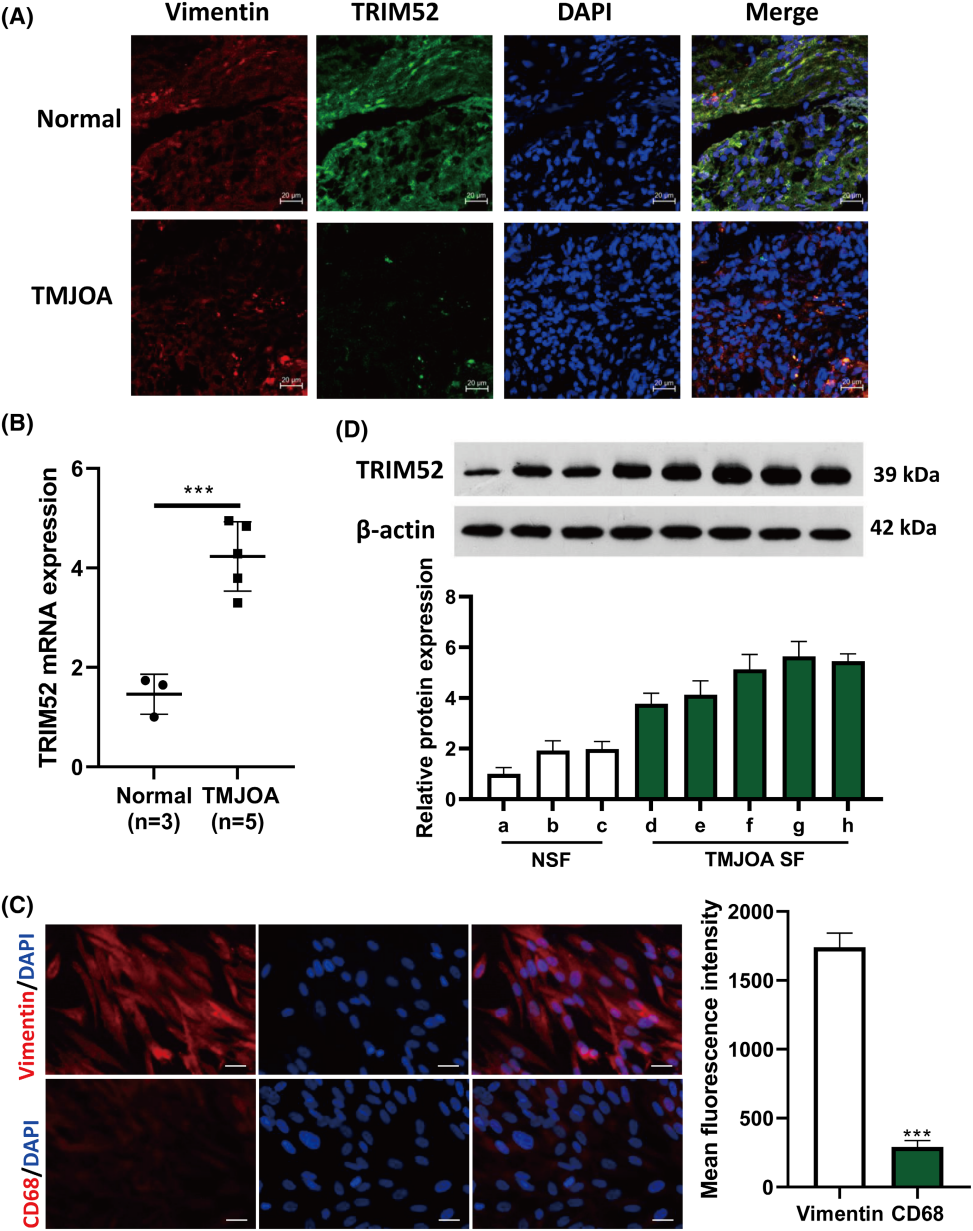
Tripartite Motif 52 (TRIM52) was upregulated in temporomandibular joint osteoarthritis (TMJOA). (A) Quantitative real‐time polymerase chain reaction was used to analyse TRIM52 expression in the synovial tissue of patients with TMJOA and the normal synovial specimens. (B) The expression of vimentin and CD68 in primary synovial fibroblasts (SFs) was determined by immunofluorescence, scale bar = 10 μm. (C) Western blot was performed to detect TRIM52 expression in TMJOA SFs (TMJOA SF‐d/e/f/g/h) and NSFs (NSF‐a/b/c). ***p < 0.001.

To clarify the function of TRIM52 in TMJOA, we collected five TMJOA synovial tissues and third normal, non‐inflamed synovial tissues and detected TRIM52 expression in synovial tissues. The results showed that the TRIM52 expression in the synovial tissue of TMJOA patients was significantly higher than that in the normal membrane tissue (*p* < 0.001, Figure 1A). Then the primary SFs were then isolated and identified by immunofluorescence staining. The isolated SFs were spindle‐shaped, with positive expression of vimentin and negative expression of CD68, and the purity exceeded 95% (Figure 1B). The above results indicated that the primary SFs were successfully isolated (Figure 1B). Western blot results also further showed that the TRIM52 protein levels were strikingly increased in TMJOA SFs (TMJOA SF‐d/e/f/g/h) relative to NSFs (NSF‐a/b/c) (Figure 1C). These findings suggested that TRIM52 might be related to TMJOA.

### 3.2 IL‐1β treatment upregulated TRIM52 expression in TMJOA SFs and NSF

**FIGURE 2:**
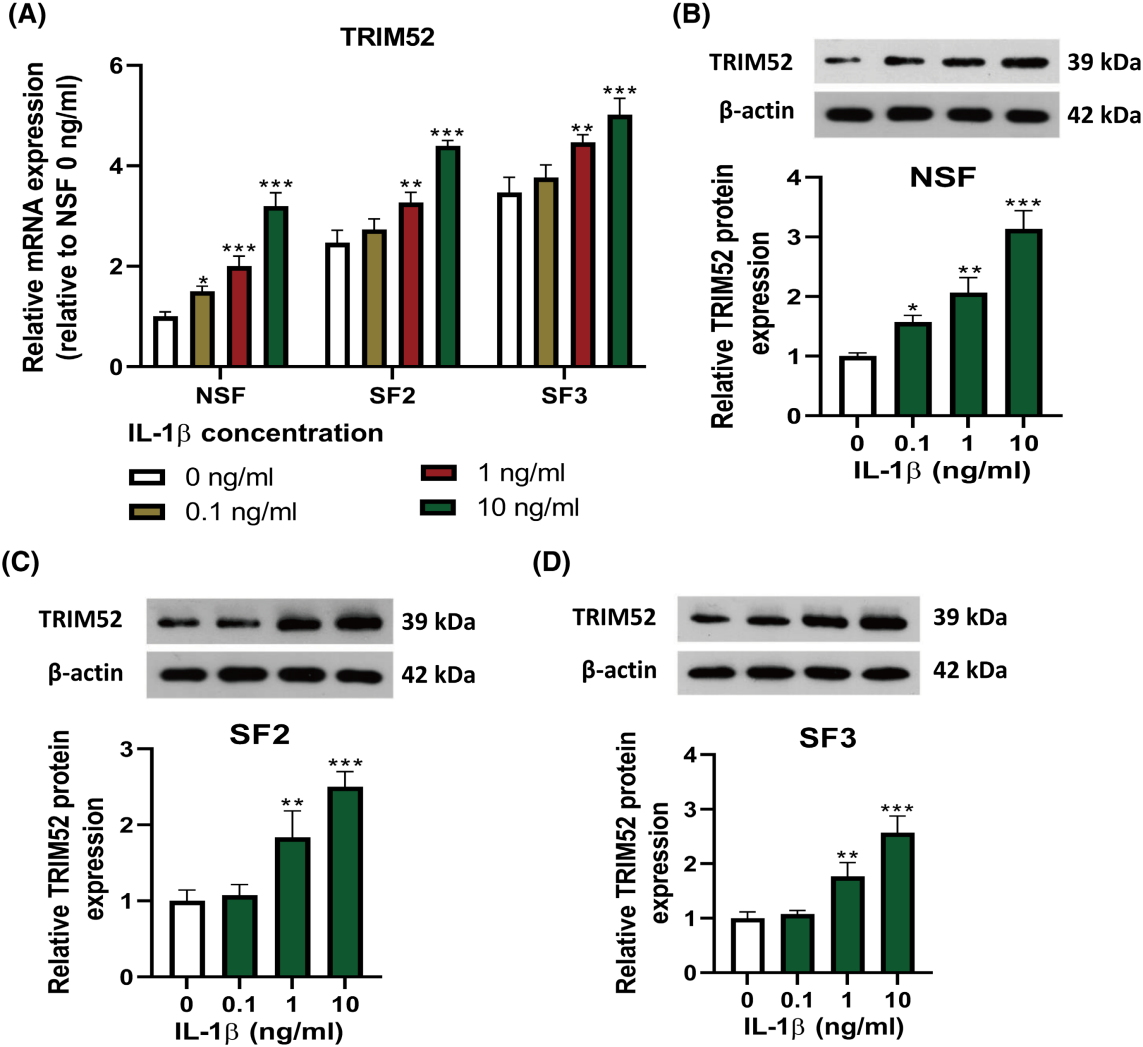
Interleukin (IL)‐1β treatment upregulated TRIM52 expression in TMJOA SF and NSF. TMJOA SF (SF2 and SF3) and NSF were seeded in a 24‐well plate and cultured for 24 h. Then all SFs were serum‐starved for 8 h and then stimulated with IL‐1β (0, 0.1, 1 and 10 ng/mL) for 24 h. The quantitative real‐time polymerase chain reaction was utilized to measure TRIM52 mRNA expression in TMJOA SFs (SF2 and SF3) and NSF (A). TRIM52 protein expression in TMJOA SFs (SF2 and SF3) and NSF (B–D) was analysed by Western blot. *p < 0.05, **p < 0.01, ***p < 0.001 versus IL‐1β 0 ng/mL group.

To more closely simulate the pathophysiology of SF in TMJOA, we treated isolated SF with IL‐1β as an inflammatory inducer. Isolated SFs were treated by different concentrations of IL‐1β (0, 0.1, 1 and 10 ng/mL) for 24 h to construct a cell model. IL‐1β administration dose‐dependently increased the mRNA and protein expression of TRIM52 in NSF, SF2 and SF3, as determined by qRT‐PCR and western blot. Notably, IL‐1β at low concentration (0.1 ng/mL) markedly elevated the mRNA and protein levels of TRIM52 in NSF, but slightly upregulated the levels of TRIM52 in SF2 and SF3 (Figure 2A–D). These results revealed that IL‐1β treatment upregulated the expression of TRIM52 in TMJOA SF and NSF in a concentration‐dependent manner.

### 3.3 Impact of TRIM52 downregulation on IL‐1β‐induced cellular function

**FIGURE 3:**
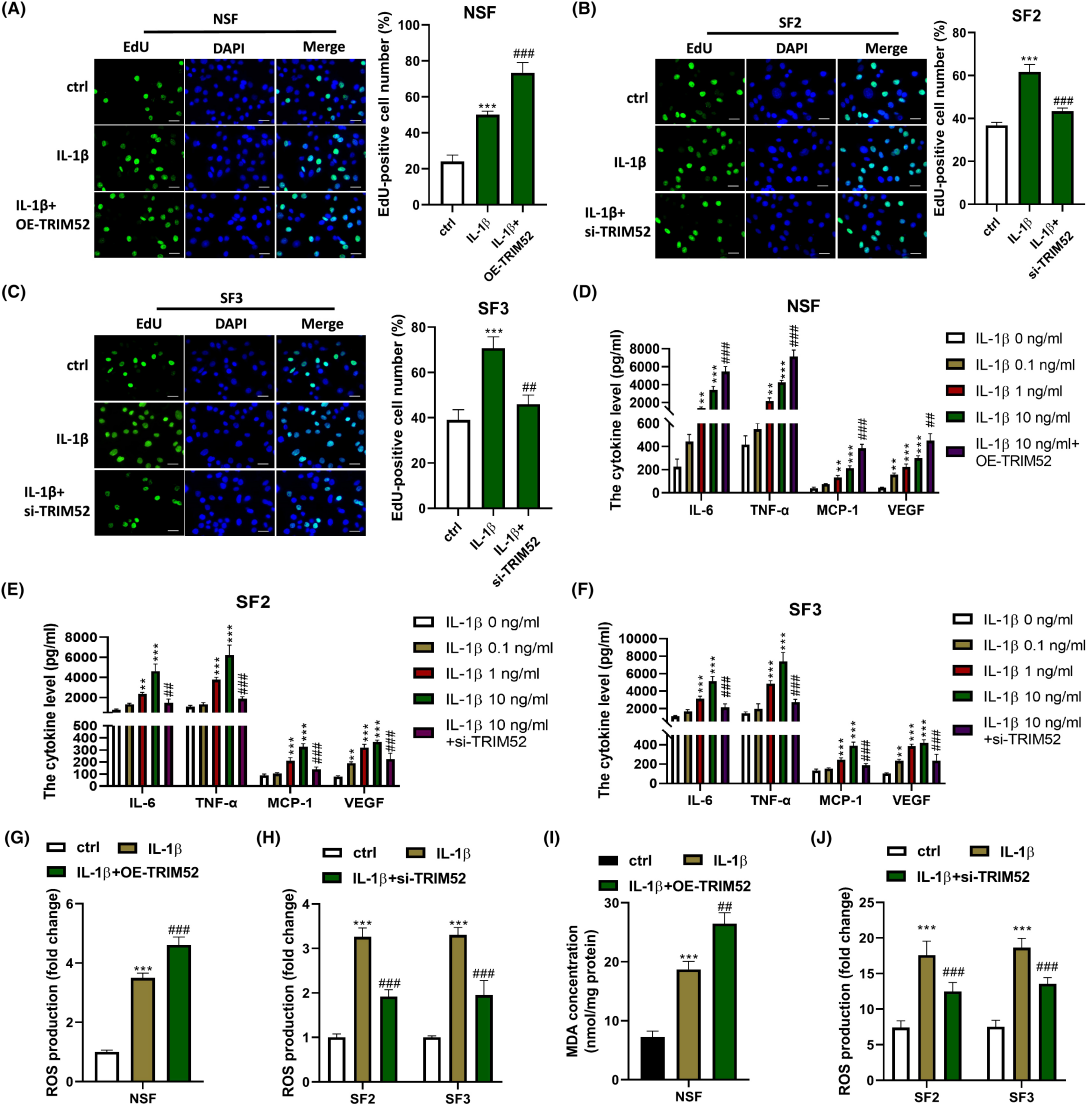
Knockdown of TRIM52 inhibited IL‐1β‐induced proliferation, inflammatory response and oxidative stress in SFs. (A–C) 5‐Ethynyl‐2′‐deoxyuridine (EdU) assay was performed to evaluate the proliferation of NSF (A), SF2 (B) and SF3 (C), scale bar = 10 μm.***p < 0.001 versus control group; ^##^ *p* < 0.01 and ^###^ *p* < 0.001 versus IL‐1β group. The levels of IL‐6, tumour necrosis factor‐alpha (TNF)‐α, monocyte chemoattractant protein‐1 (MCP‐1) and vascular endothelial growth factor (VEGF) in NSF (D), SF2 (E) and SF3 (F) were examined by enzyme‐linked immunosorbent assay (ELISA) assay. **p < 0.01, ***p < 0.001 versus IL‐1β 0 ng/mL group; ^##^ *p* < 0.01 and ^###^ *p* < 0.001 versus IL‐1β 10 ng/mL group. The reactive oxygen species (ROS, G/H) and malondialdehyde (MDA, I/J) levels in NSF and SF2, SF3 were detected. ***p < 0.001 versus control group; ^##^ *p* < 0.01 and ^###^ *p* < 0.001 versus IL‐1β group.

Since the expression of TRIM52 in SF2 and SF3 was higher than that in NSF, we knocked down TRIM52 in SF2 and SF3, and TRIM52 was overexpressed in NSF to explore the role of TRIM52. The silence and overexpression were identified by qRT‐PCR and Western blot (Figure S1A–F). As determined by the CCK‐8 assay, SF2 and SF3 were found to proliferate faster than NSF under normal conditions. IL‐1β treatment promoted the cell proliferation of NSF, SF2 and SF3 in a dose‐dependent manner. Remarkably, SF2 and SF3 were unsusceptible to the low concentration of IL‐1β. Therefore, 10 ng/mL IL‐1β was used to induce NSF, SF2 and SF3 cells in a follow‐up study. Furthermore, we found that upregulation of TRIM52 enhanced IL‐1β‐induced NSF proliferation, while the silence of TRIM52 inhibited the proliferation of NSF in the presence of IL‐1β (Table 1). In parallel, IL‐1β treatment (10 ng/mL) increased the number of EdU‐positive cells in NSF, SF2 and SF3. Overexpression of TRIM52 strengthened the proliferation of NSF triggered by IL‐1β in NSF (Figure 3A), whereas deletion of TRIM52 attenuated IL‐1β‐induced proliferation in SF2 and SF3 (Figure 3B,C).

**TABLE 1:** The effects of TRIM52 and IL‐1β on SFs proliferation by CCK‐8.

Time	Groups	NSF	SF2	SF3
0 h	All groups	1.00 ± 0.12	0.98 ± 0.13	1.01 ± 0.09
56 h	Control	1.78 ± 0.15	2.33 ± 0.25	2.81 ± 0.21
	IL‐1β (0.1 ng/mL)	2.15 ± 0.17*	2.75 ± 0.31	3.15 ± 0.39
	IL‐1β (1 ng/mL)	2.95 ± 0.35*	3.74 ± 0.42*	4.22 ± 0.34*
	IL‐1β (10 ng/mL)	5.31 ± 0.42*	6.51 ± 0.48*	7.29 ± 0.54*
	IL‐1β (10 ng/mL) + pcDNA‐TRIM52	7.81 ± 0.61**	–	–
	IL‐1β (10 ng/mL) + si‐TRIM52	–	2.98 ± 0.31**	3.25 ± 0.42**

*Note*: The data are expressed as the mean ± SEM.

* *p* < 0.05 compare with control group.

** *p* < 0.05 compare with IL‐1β (10 ng/mL) group.

To study the impact of TRIM52 on IL‐1β‐induced inflammatory response and oxidative stress, the levels of inflammatory cytokines (IL‐6, TNF‐α, MCP‐1 and VEGF) and oxidative stress substances (ROS and MDA) were detected. The results were shown in Figure 3D–J. IL‐1β induced the levels of inflammatory cytokines and oxidative stress‐related substances in NSF, SF2 and SF3 in a dose‐dependent manner. Moreover, the levels of IL‐6, TNF‐α, MCP‐1, VEGF, ROS and MDA were strongly higher in SF2 and SF3 than those in NSF. Additionally, upregulation of TRIM52 increased IL‐6, TNF‐α, MCP‐1, VEGF, ROS and MDA levels in NSF stimulated with 10 ng/mL IL‐1β, while silence of TRIM52 decreased IL‐1β‐induced IL‐6, TNF‐α, MCP‐1, VEGF, ROS and MDA levels in SF2 and SF3 (*p* < 0.001). These findings showed that TRIM52 knockdown relieved IL‐1β‐induced inflammatory response and oxidative stress in SFs.

### 3.4 TRIM52 upregulated the TLR4/NF‐κB signalling pathway in IL‐1β induced SFs

**FIGURE 4:**
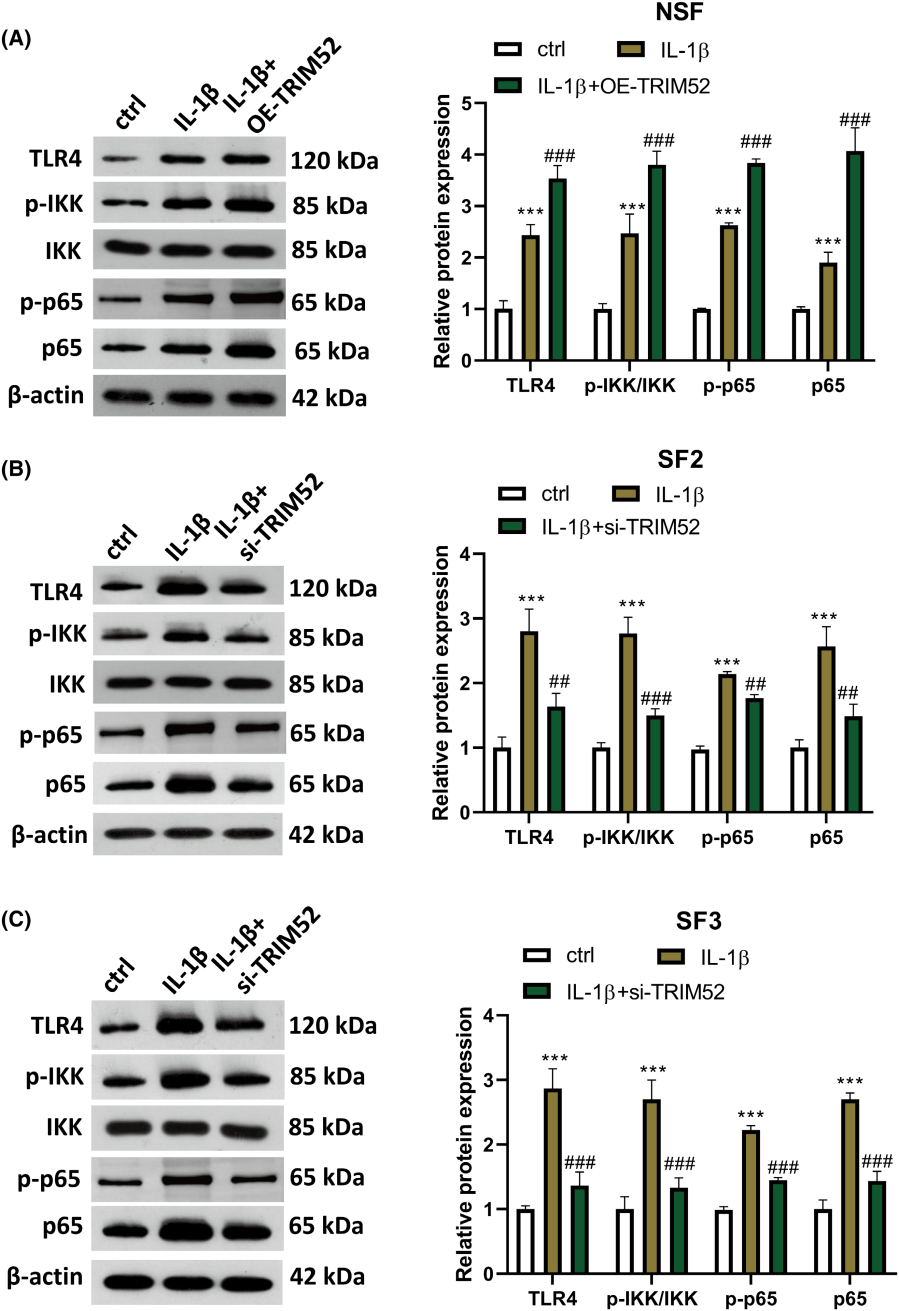
TRIM52 upregulated Toll‐like receptor 4 (TLR4)/nuclear factor (NF)‐κB signalling pathway. After treatment, NSF (A), SF2 (B) and SF3 (C) were harvested and assayed for the levels of TLR4, p‐IkappaB kinase (IKK)/IKK and p65 using western blot. *p < 0.05, **p < 0.01 and ***p < 0.001 versus control group; ^##^ *p* < 0.01 and ^###^ *p* < 0.001 versus IL‐1β group.

Previous studies have shown that the TLR4/NF‐κB signalling pathway plays a key role in the inflammatory and other pathological changes of TMJOA.^17^, ^18^ To investigate whether TRIM52 regulated IL‐1β‐induced proliferation, oxidative stress and inflammatory response via TLR4/NF‐κB signalling pathway, we detected the expression levels of TLR4/NF‐κB signalling pathway‐related proteins (TLR4, p‐IKK/IKK and p65) in IL‐1β‐induced SFs transfected with pcDNA‐TRIM52 or si‐TRIM52 using western blot. In NSF, IL‐1β treatment evidently increased the expression of TLR4, p‐IKK/IKK and p65, while TRIM52 upregulation further enhanced the following expression (Figure 4A). In SF2 and SF3, the upregulation of TLR4, p‐IKK/IKK and p65 were also discovered after IL‐1β treatment, but these changes induced by IL‐1β induction could be attenuated by knockdown of TRIM52 (Figure 4B,C). These results indicated that TRIM52 knockdown significantly inhibited IL‐1β‐induced activation of the TLR4/NF‐κB signalling pathway.

### 3.5 Pyrrolidine dithiocarbamic acid restrained cell proliferation, inflammatory response and oxidative stress in IL‐1β induced SFs

**FIGURE 5:**
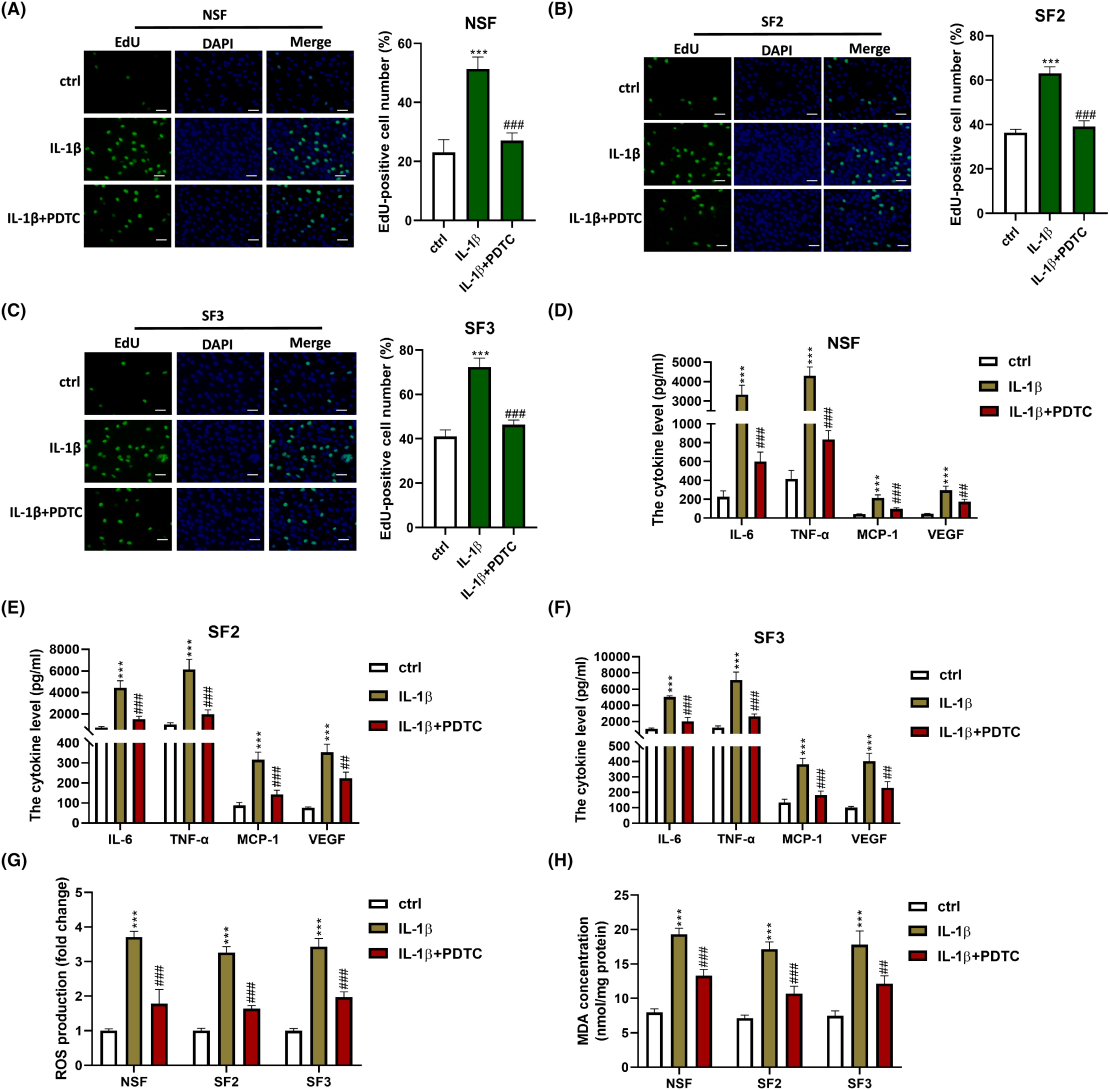
PDTC restrained cell proliferation, inflammatory response and oxidative stress in IL‐1β induced SFs. NSF, SF2, and SF3 were treated with IL‐1β alone or together with pyrrolidinedithiocarbamic acid. After 24 h of treatment, NSF (A), SF2 (B) and SF3 (C) were collected and subjected to EdU assay, scale bar = 10 μm. ELISA assay was carried out to evaluate the levels of IL‐6, TNF‐α, MCP‐1 and VEGF in NSF (D), SF2 (E) and SF3 (F). ROS (G) and MDA (H) levels was detected by biochemical kit. ***p < 0.001 versus control group; ^*##*^ *p* < 0.01 and ^###^ *p* < 0.001 versus IL‐1β group.

To further identify whether TLR4/NF‐κB signalling participates in IL‐1β induced SFs proliferation, inflammatory response and oxidative stress, NSF, SF2 and SF3 were treated with IL‐1β alone or along with 25 μM pyrrolidine dithiocarbamic acid (PDTC) (the TLR4/NF‐κB signalling pathway inhibitor). As shown in Table 2, PDTC alleviated cell proliferation in IL‐1β‐induced NSF, SF2 and SF3 by CCK‐8 assay. Consistent with this, PDTC blocked IL‐1β‐induced increases in the percentage of EdU‐positive NSF, SF2 and SF3 (Figure 5A–C). Besides, the secretion of IL‐6, TNF‐α, MCP‐1, VEGF, ROS and MDA in NSF, SF2 and SF3 was induced by IL‐1β, and such induction was attenuated following PDTC treatment (Figure 5D–H), These results suggested that PDTC restrained cell proliferation, inflammatory response and oxidative stress in IL‐1β induced SFs.

**TABLE 2:** The effects of PDTC on cell proliferation by CCK‐8 assay.

Time	Groups	NSF	SF2	SF3
0 h	All groups	1.00 ± 0.09	1.01 ± 0.14	1.02 ± 0.11
	Control	1.80 ± 0.17	2.41 ± 0.27	2.92 ± 0.23
56 h	IL‐1β (10 ng/mL)	5.52 ± 0.36*	6.76 ± 0.38*	7.51 ± 0.55*
	IL‐1β (10 ng/mL) + PDTC	2.22 ± 0.25**	2.81 ± 0.31**	3.32 ± 0.35**

*Note*: The data are expressed as the mean ± SEM.

* *p* < 0.05 compare with control group.

** *p* < 0.05 compare with IL‐1β (10 ng/mL) group.

### 3.6 TRIM52 knockdown inhibited cell proliferation, oxidative stress and inflammatory response in IL‐1β induced SFs by downregulating TLR4

**FIGURE 6:**
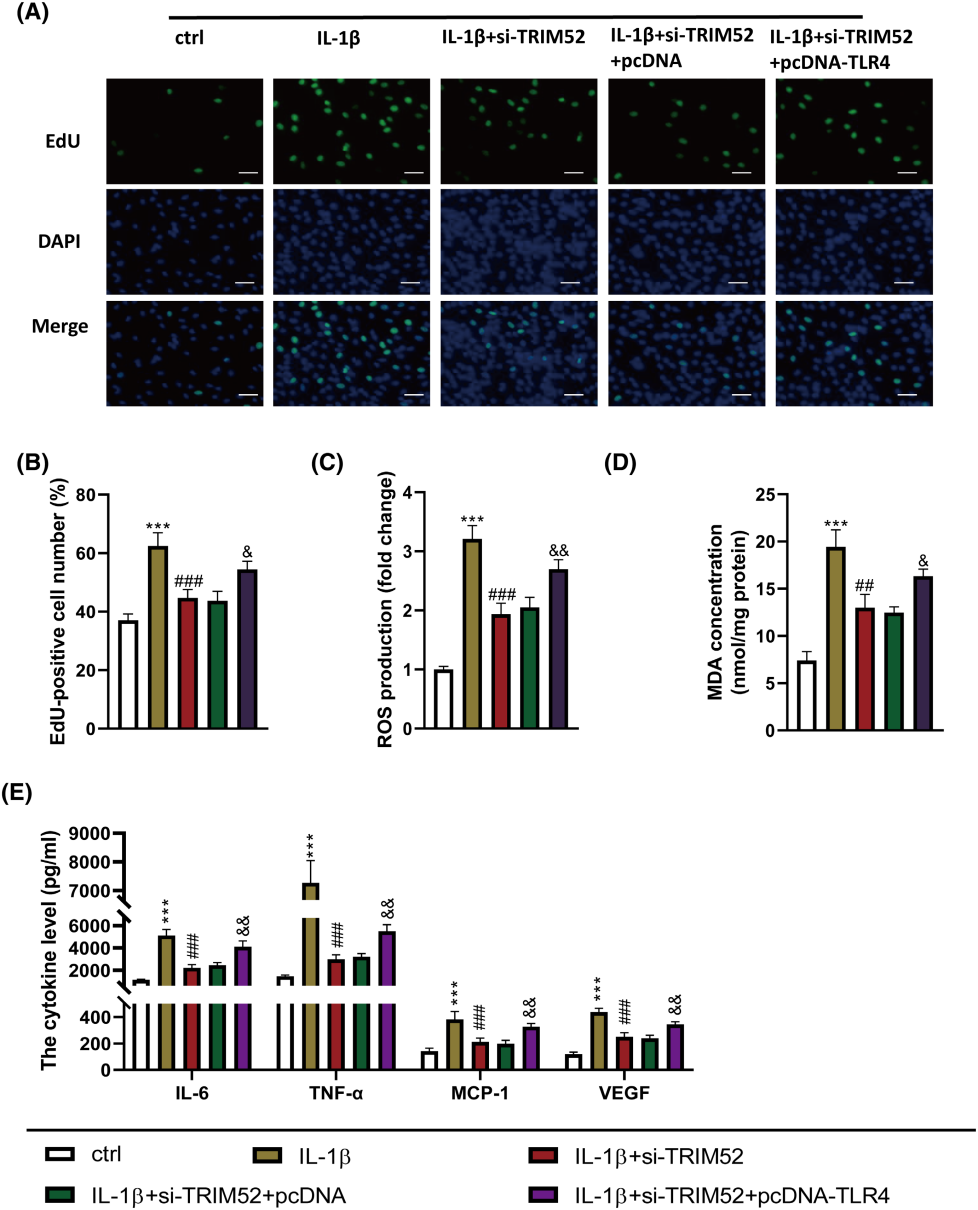
TRIM52 knockdown inhibited cell proliferation, oxidative stress and inflammatory response in IL‐1β induced SFs by downregulating TLR4. EdU assay (A) was performed to evaluate the proliferation of SFs, scale bar = 10 μm. The levels of oxidative stress factors ROS (B) and MDA (C) and inflammatory cytokines IL‐6, TNF‐α, MCP‐1 and VEGF (D) were detected. ***p < 0.001 versus control group; ^##^ *p* < 0.01 and ^###^ *p* < 0.001 versus IL‐1β group; ^&^ *p* < 0.05 and ^&&^ *p* < 0.01 versus IL‐1β + si‐TRIM52 + pcDNA group.

To determine whether TRIM2 promoted cell proliferation, inflammatory response and oxidative stress in IL‐1β‐induced SFs by regulating TLR4/NF‐κB signalling pathway, we simultaneously transfected si‐TRIM52 and pcDNA‐TLR4 into IL‐1β‐induced SFs. The levels of cell proliferation, inflammatory cytokines and oxidative stress factors were detected. Consistent with the previous results, IL‐1β promoted the proliferation and increased the levels of inflammatory cytokines and oxidative stress factors in SFs cells, while the knockdown of TRIM52 significantly inhibited these effects of IL‐1β. More importantly, in IL‐1β‐induced SFs, TLR4 overexpression significantly increased the percentage of EdU‐positive cells. Besides, the levels of ROS, MDA, IL‐6, TNF‐α, MCP‐1 and VEGF were inhibited by TRIM52 knockdown (*p* < 0.001, Figure 6A–D). The above findings indicated that TRIM52 knockdown inhibited cell proliferation, oxidative stress and inflammatory response in IL‐1β‐induced SFs by downregulating TLR4.

### 3.7 TRIM52 knockdown relieved the pathological damage of synovial and cartilage tissues in the TMJOA rat by downregulating TLR4

**FIGURE 7:**
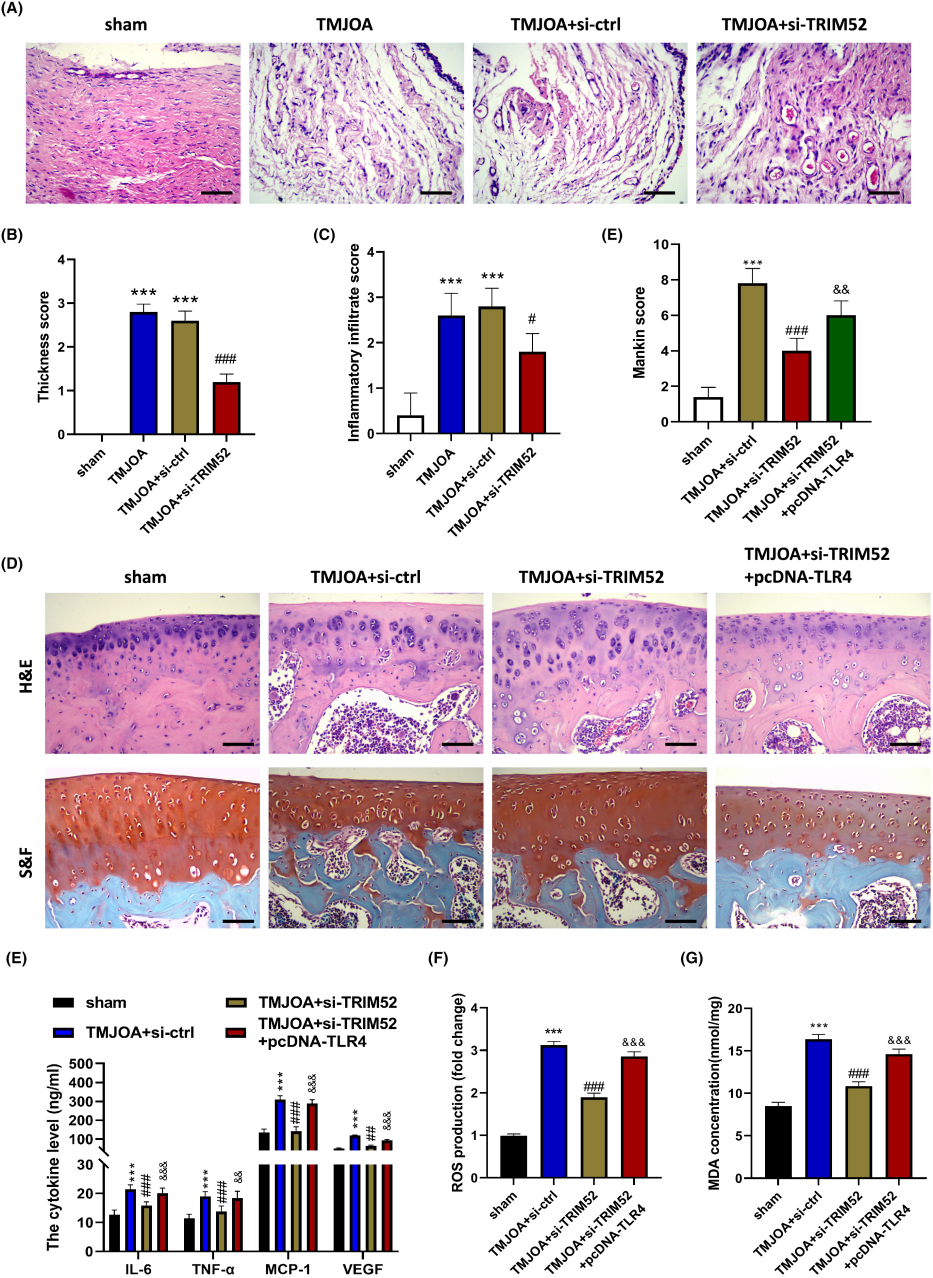
TRIM52 downregulation relieved synovial hyperplasia and inflammatory infiltration in the TMJOA rat model. Haematoxylin and eosin was used to stain photomicrographs of synovial tissue in TMJOA rats of different treatment groups (A) to analyse the thickness score (B) and inflammatory infiltration score (C). ***p < 0.001 compare with the sham group; ^#^ *p* < 0.05 and ^###^ *p* < 0.001 compare with TMJOA + si‐ctrl group. Haematoxylin and eosin and safranin O/fast green were performed to stain photomicrographs of cartilage tissues in rats (E) to analyse Mankin score (D), scale bar = 50 μm. The levels of inflammatory cytokines (IL‐6, TNF‐α, MCP‐1 and VEGF, E) and oxidative stress factors ROS (F) and MDA (G) were analysed. ***p < 0.001 compare with the sham group; ^###^ *p* < 0.001 compare with the TMJOA + si‐control group; ^&&&^ *p* < 0.001 and ^&&^ *p* < 0.01 compare with the TMJOA + si‐TRIM52 group. The data were expressed as the mean ± standard error of mean for five rats per group.

The occlusion disorder could cause abnormal direction and distribution of the occlusal force, resembling the pathogenesis of human osteoarthritis. Occluse‐induced osteoarthritis is thus regarded as a suitable model to analyse pathogenesis. Here, to explore the functions of TRIM52 in vivo, we constructed a TMJOA rat model through occlusal induction and inhibited TRIM52 expression in TMJOA rats by injection of si‐TRIM52. Haematoxylin and eosin staining results were shown in Figure 7A–C. Compared with the sham group, the synovial tissue of rats in the TMJOA group and TMJOA + si‐ctrl group was significantly thickened and the inflammatory infiltration was more serious (*p* < 0.001). Such results suggested that the TMJOA rat model was successfully constructed. The thickness and inflammatory infiltration score of synovial tissue in the si‐TRIM52 group were significantly decreased compared with the TMJOA + si‐ctrl group (*p* < 0.05).

In addition, haematoxylin and eosin, and S&F staining results showed that compared with the sham group, the articular cartilage layer was thinner, proteoglycan and chondrocytes were significantly reduced in the TMJOA + si‐ctrl group, and cartilage tissue was degraded. After TRIM52 knockdown, the chondrocytes and proteoglycans of TMJOA rats increased significantly, while the overexpression of TLR4 reversed the relief effect of si‐TRIM52 on the histopathologic injury of TMJOA rats (Figure 7D). Similarly, Mankin score results also proved that Mankin score in the TMJOA group was increased as opposed to the sham group, indicating the degeneration of articular cartilage (*p* < 0.001). Furthermore, si‐TRIM52 treatment significantly reduced the Mankin score of TMJOA (*p* < 0.001), and this effect was reversed by TLR4 overexpression (*p* < 0.01, Figure 7E). These findings suggested that TRIM52 knockdown relieved the pathological damage of synovial and cartilage tissues in the TMJOA rat by downregulating TLR.

## 4 DISCUSSION

Although the pathogenesis of TMJOA remains poorly understood to date, inflammation and oxidative stress have been recognized as the main contributors to the pathogenesis of TMJOA.^6^, ^19^ The overproduction of ROS contributes to synovial inflammation and dysfunction of the subchondral bone in OA.^20^ Previously, the elevation of pro‐inflammatory cytokines has been documented to play a vital role in the progression of TMJOA.^21^ Among these inflammatory cytokines, IL‐1β is recognized as one of the major pro‐inflammatory mediators of TMJOA progression.^22^ It was widely established that the upregulation of IL‐1β is a distinctive characteristic of TMJOA.^23^ IL‐1β can induce the generation of extracellular matrix‐degrading enzyme and chemokines, leading to arthritis and cartilage and bone destruction. Such process can result in TMJOA.^24^ Also, IL‐1β induces the secretion of IL‐6 and then impedes the chondrogenic differentiation of synovial fluid mesenchymal stem cells in the temporomandibular joint.^25^ Therefore, restricting IL‐1β‐induced inflammatory response and oxidative stress in SF may be a potential approach for the treatment of TMJOA. In this study, we used IL‐1β as a stimulator to mimic the progression of TMJOA in vitro, and found that IL‐1β treatment promoted cell proliferation, inflammatory response and oxidative stress in NSF, SF2 and SF3.

RIM family proteins play a regulatory role in a variety of physiological and pathological processes, however, fewer researches on the roles of TRIM52 have been elucidated to date.^26^, ^27^ Recently, several studies have associated TRIM52 with the progression of human cancers.^28^ For example, TRIM52 has been reported to interact with Src homology two domain‐containing protein tyrosine phosphatase‐2 to promote the ubiquitination of SHP2 and the activation of signal transducer and activator of transcription 3 signalling is induced. Ultimately, such process promotes the proliferation and inhibits the apoptosis of human colorectal cancer cells in vitro and in vivo.^29^ In addition, targeting TRIM52 can block cell cycle progression and inhibit cell proliferation and invasiveness by inducing the inactivation of the Wnt/β‐catenin signalling pathway.^30^ In hepatocellular carcinoma, forced expression of TRIM52 promotes the proliferation, migration and invasion of MHCC‐97 L cells by inhibiting dephosphorylation of Smad 2/3 through inducing the ubiquitination of phosphatase magnesium‐dependent 1A.^31^ Besides, TRIM52 reportedly promotes hepatitis B virus‐induced the fibrogenesis of human hepatic stellate cell line LX‐2 cells through phosphatase magnesium‐dependent 1A‐mediated transforming growth factor‐β/Smad signalling.^32^ TRIM52 is upregulated in hepatitis B virus‐associated hepatocellular carcinoma, and the upregulation of HBV X protein induces the activation of NF‐κB signalling to increase the expression of TRIM52 in HepG2 cells, which in turn promotes the proliferation of HepG2 cells.^33^ However, the role of TRIM52 in the progression of TMJOA has not yet been explored to date. In this study, the upregulation of TRIM52 was found in the synovial tissue of patients with TMJOA, TMJOA SFs as well as IL‐1β‐induced SFs. Silence of TRIM52 inhibited IL‐1β‐induced proliferation, inflammatory response and oxidative stress in SF2 and SF3, while forced expression of TRIM52 enhanced IL‐1β‐induced proliferation, inflammatory response, and oxidative stress in NSF. Additionally, in vivo study also confirmed that the knockdown of TRIM52 inhibited synovial hyperplasia and inflammatory infiltration. Although the function of TRIM52 in OA has not been studied, a previous study also showed that TRIM52 knockdown alleviates LPS‐induced inflammatory damage to human periodontal ligament cells.^12^ However, there are no studies on the effect of TRIM52 on oxidative stress in diseases, and this study revealed the relationship between the downregulation of TRIM52 and oxidative stress in diseases for the first time. These findings revealed that targeting TRIM52 might be a promising approach for TMJOA therapy.

Although the role of TRIM52 has been clarified in our study, the exact mechanism of action is still needed to investigate. TLR4 is a transmembrane receptor that is essential for the activation of all sorts of signalling pathways in the host immune response.^34^, ^35^ Accumulating evidence has shown that TLR4/NF‐κB signalling plays a principal role in the progression of TMJOA. In the patients with TMJOA, the expression of TLR4 and NF‐κB p65 is increased, and inhibition of TLR4 by TAK‐242 can decrease the generation of MyD88/NF‐κB, pro‐inflammatory and catabolic mediators in IL‐1β‐stimulated chondrocytes. The destruction of cartilage and subchondral bone can be alleviated in discectomy‐induced TMJOA mice.^36^ Notably, the involvement of TRIM52 in the activation of TLR4/NF‐κB signalling has been reported in the last years. TRIM52 has been identified to function as a vital regulator of inflammation. Previously, TRIM52 has been considered as a positive modulator of NF‐κB signalling.^37^ Ectopic expression of TRIM52 promotes the ubiquitination of inhibitory kappa B‐alpha to activate the NF‐κB signalling, which in turn enhances lipopolysaccharide‐induced microglial cell activation and the inflammatory response.^38^ Besides, activation of NF‐κB signalling has been demonstrated to be implicated in TRIM52‐mediated tumorigenesis.^28^ However, if the activation of TLR4/NF‐κB signalling pathways is involved in TRIM52‐mediated TMJOA progression is still elusive. In the present study, we found that PDTC ameliorated IL‐1β‐induced proliferation and inflammatory response by inhibiting TLR4/NF‐κB signalling pathways, revealing the participation of TLR4/NF‐κB signalling in TMJOA progression. Further research confirmed that IL‐1β treatment upregulated the activity of TLR4/NF‐κB pathway. Besides, IL‐1β‐induced activation of the TLR4/NF‐κB signalling was enhanced by upregulation of TRIM52 in NSF, and was attenuated by silence of TRIM52 in SF2 and SF3. In addition, TLR4 overexpression could reverse the inhibitory effects of TRIM52 knockdown on SFs proliferation, inflammatory response, and oxidative stress in TMJOA in vivo or in vitro. The above results indicated that TRIM52 regulated IL‐1β‐induced inflammatory response via the TLR4/NF‐κB pathway.

However, as a preliminary study, this study has some limitations. Although this study demonstrated that TRIM52 knockdown played a role in inhibiting cell proliferation, inflammation, and oxidative stress in TMJOA in vivo and in vitro through the TLR4/NF‐κB signalling pathway, its effects on signalling pathways remain unclear. Meanwhile, we only evaluated the function of TRIM52 by histopathological staining in vivo experiments, and there was a lack of quantitative studies on related factors. In addition, it is not clear whether TRIM52 plays a role in bone differentiation. Therefore, a large number of studies need to be carried out to clarify the effect of TRIM52 on TMJOA and its mechanism.

## 5 CONCLUSION

In summary, TRIM52 was found to be upregulated in the synovial tissue of patients with TMJOA, as well as TMJOA SFs. Knockdown of TRIM52 inhibited proliferation, inflammatory responses and oxidative stress in IL‐1β induced SFs by regulating TLR4/NF‐κB pathway. This study manifested that targeting TRIM52 might be a potential approach for the treatment of TMJOA.

## AUTHOR CONTRIBUTIONS

**Tie Ma:** Conceptualization (equal); investigation (equal); software (equal). **Chuan‐bin Wu:** Conceptualization (equal); methodology (equal); software (equal). **Qing‐xia Shen:** Data curation (equal); formal analysis (equal). **Qiang Wang:** Data curation (equal); formal analysis (equal). **Qing Zhou:** Investigation (equal); project administration (equal); writing – original draft (equal).

## FUNDING INFORMATION

None.

## CONFLICT OF INTEREST STATEMENT

The authors declare no competing interests.

## Supporting information


**Figure S1.** Construction of TRIM52 overexpression/knockdown SFs. TRIM52 was overexpressed and knocked down in NSF, SF2, and SF3, respectively. A–F, Validation of the TREM52 mRNA and protein expression levels in transfected NSF (A/B), SF2 (C/D) and SF3 (E/F) were detected by RT‐qPCR and western blot. **p < 0.01 and ***p < 0.001 versus pcDNA‐control or si‐control.

**Table S1.** Sequence of the siRNA fragment.

## Data Availability

The datasets used and/or analyzed during the current study are available from the corresponding author on reasonable request.
