# Supplementary figures and images for: TRIM52 knockdown inhibits proliferation, inflammatory responses and oxidative stress in IL‐1β‐induced synovial fibroblasts to alleviate temporomandibular joint osteoarthritis
Source: J Cell Mol Med. 2024 Mar 23;28(8):e18244. doi: 10.1111/jcmm.18244 (PMC10960171; doi:10.1111/jcmm.18244)

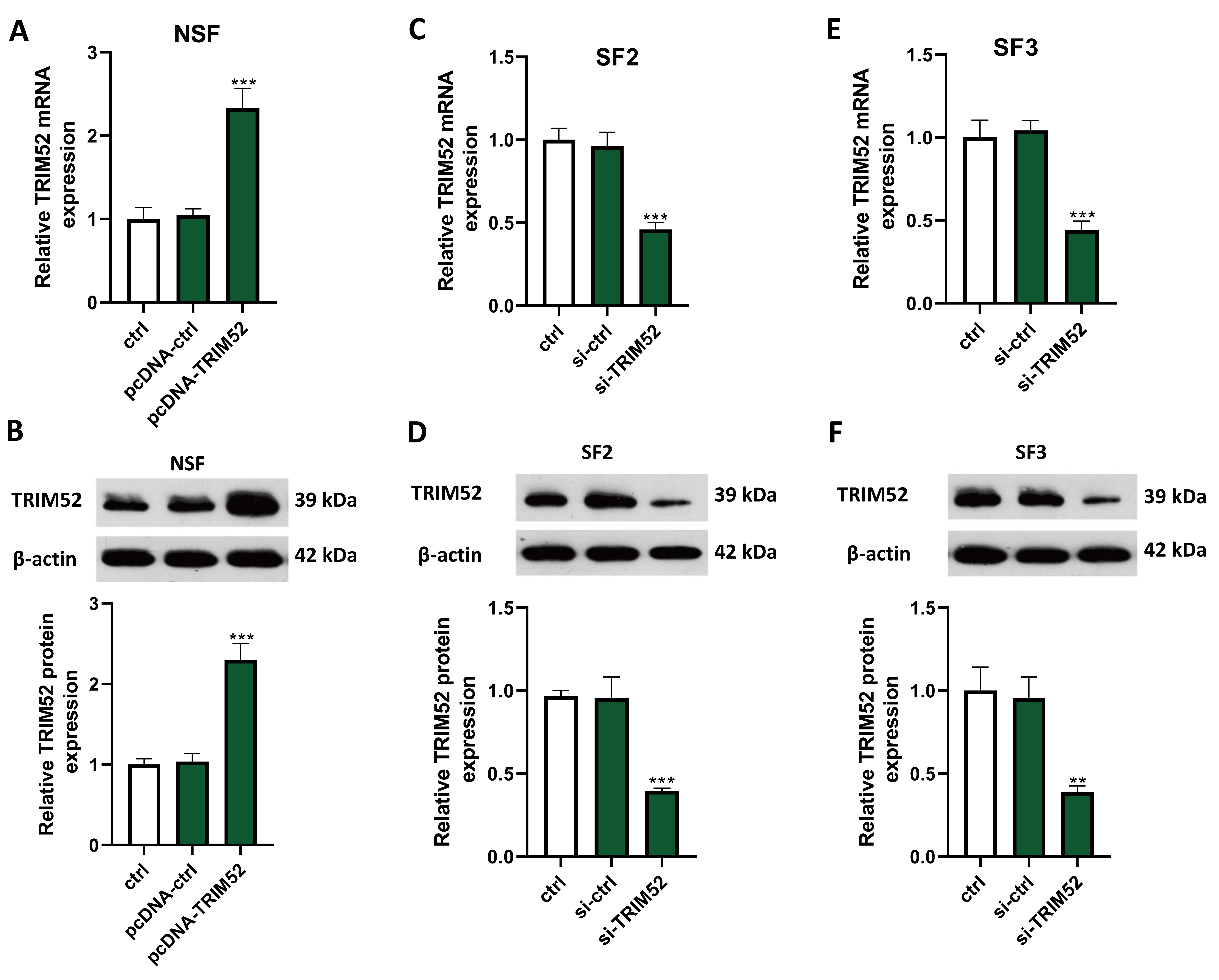

Supplement: Supplementary file 1 — Figure S1. Construction of TRIM52 overexpression/knockdown SFs. TRIM52 was overexpressed and knocked down in NSF, SF2, and SF3, respectively. A–F, Validation of the TREM52 mRNA and protein expression levels in transfected NSF (A/B), SF2 (C/D) and SF3 (E/F) were detected by RT‐qPCR and western blot. **p < 0.01 and ***p < 0.001 versus pcDNA‐control or si‐control. [file JCMM-28-e18244-s001.tif]
