# Supplementary material for: TRIM52 knockdown inhibits proliferation, inflammatory responses and oxidative stress in IL‐1β‐induced synovial fibroblasts to alleviate temporomandibular joint osteoarthritis
Source: J Cell Mol Med. 2024 Mar 23;28(8):e18244. doi: 10.1111/jcmm.18244 (PMC10960171; doi:10.1111/jcmm.18244)
Supplement: Supplementary file 2 — Table S1. Sequence of the siRNA fragment. [file JCMM-28-e18244-s002.docx]

| **Species: name** | **Sequences** |
| --- | --- |
| Rat: siRNA ctrl | Sense Sequence: ACACCAGGGCAACGCGUCUCGGGUA  Antisense Sequence: UACCCGAGACGCGUUGCCCUGGUGU |
| Rat: siTRIM52 | Sense Sequence: ACAGGACCGGGAACCGCCUGCUGUA  Antisense Sequence: UACAGCAGGCGGUUCCCGGUCCUGU |
| Human: siRNA ctrl | Sense Sequence: ACACCAGGGCAACGCGUCUCGGGUA Antisense Sequence: UACCCGAGACGCGUUGCCCUGGUGU |
| Human: siTRIM52 | Sense Sequence: ACAGGACCGGGAACCGCCUGCUGUA Antisense Sequence: UACAGCAGGCGGUUCCCGGUCCUGU |

**Supplementary table 1** Sequence of the siRNA fragment.
